# Multiple Lurias. Reconstructing Alexander Romanovich’s Life-Writing

**DOI:** 10.1007/s12124-024-09872-6

**Published:** 2025-01-25

**Authors:** Carlos Kölbl, Alexandre Métraux

**Affiliations:** 1 Lehrstuhl für Psychologie, Kulturwissenschaftliche Fakultät, Universität Bayreuth, Bayreuth, 95440 Germany; 2 Archives Henri Poincaré, Université de Lorraine, Nancy, France

**Keywords:** Luria studies, Life-writing, Cultural-historical psychology, Soviet psychology, History of science

## Abstract

While widely considered Alexander Luria’s (1902–1977) autobiography, *The Making of Mind. A Personal Account of Soviet Psychology*, published posthumously in 1979, is not a true autobiography but rather an *autobiography with heterobiographic elements*. However, the largely overlooked Spanish book, *Mirando hacia atrás. La vida de un psicólogo soviético en retrospección*, published in the same year, may be regarded as an authentic autobiography written by Luria. Based on the close reading of previously unknown archival sources, including Luria’s autobiographic typescripts, the central argument of the article shows that it is likely that the history of this key figure in modern Soviet neuropsychology is embodied in various instances of his life-writing. Our reconstruction of Luria’s life-writing is transnational in scope and multi-language based. It aims at drawing an overall account, which may later be followed by a series of contributions dealing with details of the history of Luria’s life-writing.

“El original es infiel a la traducción …”

(The original is unfaithful to the translation…)

Jorge Luis Borges, Obras completas, I, p. 732.

## Introduction: Some Irritations

Alexander Romanovich Luria (1902–1977), a brilliant and productive scientist, certainly ranks among the best known and highly recognised Soviet psychologists internationally. While considered one of the founders of neuropsychology and neurolinguistics, he also contributed to both cultural and developmental psychology. Furthermore, Luria promoted the work of his late friend Lev S. Vygotsky (1896–1934).

All this is already more or less known.

Public knowledge about Luria’s life and work rests largely on what are taken to be his autobiographic writings, although his daughter Elena (E. Luria [Lurija],^1^,^1991^, ^1994^), Karl Levitin (Levitin, 1998) and Evgenia D. Homskaya (Homskaya, 2001) also published their recollections of his life.^2^

However, Luria’s autobiography is hardly *but one single* or undisputed “self-made self-portrait”, a slightly awkward expression that we use for semantic reasons explained below. The issues inherent in the qualification “being self-made” ascribed to Luria’s self-portrait in prose lie at the centre of this article.

Our thesis is that *not one* of the *published* texts conventionally taken as Luria’s autobiographies (with two brief exceptions) turns out after close consideration to be free of more or less heterobiographic features. In other words, Luria is *not* the sole author of the books usually represented as his autobiography. We base this thesis on various publications, unpublished (i.e. archival) typescripts, correspondence, and personal communications with first-hand witnesses including Michael Cole, James Wertsch, Luciano Mecacci, Maria Serena Veggetti, and Giuseppe Cossu.

Let us begin with some irritations.

We only recently came across the Spanish book *Mirando hacia atrás. Obra postuma. La vida de un psicólogo soviético en retrospección* (Luria, 1979a) and were surprised by the statement in its prologue that this publication was the very first appearance of Alexander R. Luria’s autobiography *worldwide* (Barraquer-Bordas, 1979, p. 1). We had believed, like most people interested in Luria, that this had been *The Making of Mind. A Personal Account of Soviet Psychology* (Luria, 1979b), edited by Michael and Sheila Cole and published by Harvard University Press in the same year.

Our surprise increased as we read the Spanish book. We firmly expected it to be a translation of the Cole edition and were surprised to find that this was not the case. Instead, we had to concede that *Mirando hacia atrás* had been translated from an English-language typescript dated March 1976. Since one of us – Alexandre Métraux – had already worked on Luria’s life-writing (Métraux, 2021, pp. 173–187), we also noticed that its source greatly resembled – but was far from identical with – still another typescript that Michael Cole had sent a copy of to Métraux years earlier. This second typescript, entitled *Looking Back. The Life of a Soviet Psychologist in Retrospe*ct, had been dated March, 1977, by Luria himself. Either these dates contradicted the conventional chronology or the Spanish translator was working from something other than the original 1977 typescript that Cole had received from Luria and forwarded to VAAP,^3^ the Moscow-based Soviet copyright agency.

Had the international scientific community then become aware of the Spanish translation of Luria’s truly authentic autobiography (which had come into existence without heterobiographic elements), the remainder of the present paper would most likely be much shorter, if it were at all regarded as historiographically useful to begin with. But the fact is that Luria’s life-writing was pursued by himself and by other heterobiographically contributing persons as if the Spanish edition had never been published.

Intrigued by this discovery and hoping to rectify the established misconceptions, we resolved to find other available autobiographic texts by Luria and to closely read the transnational sources we had already found. In particular, we were intensely interested in locating the 1976 typescript. As we gathered additional material, it became clear to us that *not one* but several autobiographic portraits of Luria existed. Hence, we were dealing with a multiplicity of *Lurias*. The present paper aims to analyse this multiplicity. Due to the large amount of material we have collected, at present we are limiting ourselves to sketching the broader picture. Further in-depth analyses are likely to follow, as mentioned above.

Recent years have seen a considerable increase in the publication of transnational studies revising published and unpublished work by Soviet psychologists in the tradition of the cultural-historical school (or trend, or line of thought) and especially that of Vygotsky (e.g. Yasnitsky & van der Veer, 2015). The publication of some of his writings, such as his notebooks, for the first time has also received renewed attention (Vygotsky, 2018; for a discussion of the notebooks cf. Kölbl & Métraux, 2021).

Some new historical research on the cultural-historical school of Soviet psychology has been intended to initiate what has somewhat over-dramatically been called a “revisionist revolution”. We reject this sensationalist label, but do believe that the present study qualifies as a significant (although certainly not “revolutionary”) revision by showing that more than one author was involved in writing some versions of Luria’s autobiography, which thus turn out to be more or less heterobiographic in both nature and outlook. To be sure, Luria is internationally recognized as a pioneering cultural and neuropsychologist. There is thus no need to idealize this one scientific *persona* by way of an examination of his life-writing, nor do we feel the slightest propensity for a hagiographic revision of Luria activities as a Soviet scholar and close friend of Lev Vygotsky alive or dead.

## An Overview of Archival Texts and Publications

We now list the texts of Luria’s life-writing that we will refer to here.

What could be called “authorial narrative descriptions” of Luria’s life do exist, and not only as monograph-length publications. He wrote one article in Russian for the UNESCO journal *International Social Science Journal*, which also published English and French translations (Luria, 1973). A second similar text was published as a chapter in the prestigious *History of Psychology in Autobiography* (Luria, 1974a) in an English translation by Michael Cole; however, this was originally written in 1964. The UNESCO text was also included in a collection of Luria’s neuropsychological and neurolinguistic contributions in Italian translation (Luria [Lurija], 1974b).^4^

The 1974 printed version of Luria’s self-presentation originated in a letter Edwin G. Boring sent to Moscow in early 1964. The chair of the editorial committee of the already-famous series *History of Psychology in Autobiography* informed Luria of this official request for an autobiographic chapter on himself to be published with those of fourteen other authors as Volume V of the series. But although Luria welcomed and highly appreciated the invitation, he did not hastily accept it but tried to negotiate: he would submit his own autobiography on the condition that his close friend Alexei Leontiev, as well as his colleague Boris Teplov (a celebrated Soviet expert in differential psychology and a member of the Academy of Pedagogical Sciences of the Russian Soviet Federative Socialist Republic) also be invited, as three preeminent representatives of Soviet psychological research at the time. Rather than rejecting Luria’s proposal (or suggestion), Boring responded diplomatically, mentioning that although he had consistently voted for Leontiev the other members of the committee did not favour including him since they did not know his work; as for Teplov, they did not even recognise his name. (Admittedly, this last was also true of Boring himself). In his response, Luria played down his enthusiasm for writing the chapter while continuing to insist that his friend Leontiev and colleague Teplov still be part of the enterprise. In the end, Luria accepted the original invitation and informed Boring in August 1964 that he had used his summer vacation to complete the manuscript in Russian. Meanwhile, Volume V of the *History of Psychology in Autobiography* series had been reorganised due to Luria’s suggestions of Leotiev and Teplov, and Luria’s contribution was moved to Volume VI, scheduled for 1970 but not, in the event, published until 1974. The typescript is entitled “Fragments from / the History of Soviet Psychology / (An Essay Analysing the Stages Gone Through) / (Written for ‘The History of Psychology / in Autobiographies’ [sic]).”^5^

The only genuinely authorial book-length version of Luria’s life-writing exists as two typescripts that were from the outset written in English. The first, called *Looking Back*, is dated March 1976 but was most likely completed later. The other, a revised version of the 1976 typescript with the same title, was probably completed in March 1977 and sent to Michael Cole, who substantially reworked it.^6^ This edited version was eventually published by Harvard University Press as *The Making of Mind*, a title of the published book suggested by Arthur Rosenthal, the press’s director (E. Luria [Lurija], 1994, pp. 210–211; on Rosenthal cf. Vitello, 2013).

Luria’s authentic autobiography (sticking, for the time being, to conventional terminology) has never been published, but it appeared posthumously as edited (reworked) books; in book-length translations (of the 1976 typescript or of the Cole edition of *The Making of Mind*); and in English, Spanish, Russian, French, Italian, Brazilian Portuguese, and German. These include two reworked – and thus coauthored – English versions: *The Making of Mind* (Luria 1979b), and *The Autobiography of Alexander Luria: A Dialogue with The Making of Mind*, edited (that is, reworked) by Cole and Levitin (Luria 2006). The latter contains significantly more material than the former.

The Spanish version of the so-called autobiography was based on the March 1976 typescript. This book, published in Madrid, bears the title *Mirando hacia atrás*, which closely matches the title chosen by Luria for his own life-writing.

In the Soviet Union, Luria’s self-portrait in prose appeared three years after the publication of its US and Spanish counterparts (Luria [Lurija], 1982). This Russian version was edited by Luria’s former close assistant Evengia D. Homskaya and closely corresponds to *The Making of Mind* but with the title *Etapy proedennogo puti*, which roughly translates as *Stages Gone Through*. A second, unaltered, edition was published in the Russian Federation in 2001. One more Soviet publication of *Etapy* exists, a French translation distributed worldwide by the Moscow-based foreign-language publishing house Progress Publishers (Luria, 1985).

A Brazilian edition of the autobiography was published in São Paolo in honour of the ninetieth anniversary of Luria’s birth (Luria, 1992). This is a direct translation of the US version, including Cole’s introduction and epilogue; however, no information concerning the English source was provided. A 2017 reprint does not mention Cole’s contribution to *The Making of Mind*, either.

In 1993, a German version was published (Luria [Lurija], 1993), which one of us, Alexandre Métraux, translated from the 1982 Russian edition. This also includes an essay by Oliver Sacks on the topic of romantic science.

The Italian publication history of Luria’s self-portrait is rather peculiar. There are three books, two of which differ substantially from each other. The first, edited by Maria Serena Veggetti (Luria [Lurija], 1983; Veggetti, 1983), is according to the editor based on two sources, a March 1976 typescript by Luria entitled *Looking Back* that probably is the same typescript the Spaniards had been using (or at least a very similar one), and a Russian text, with the same main title as Homskaya’s but a different subtitle: the 1982 Russian publication has *Nauchnaya avtobiographiya* (“Scientific Autobiography”), while Veggetti cites one subtitled *Iz zapisok sovetskogo psihologa* [sic] (“From Notes of a Soviet Psychologist”). Moreover, the Russian text she cites much more closely resembles *Looking Back* than it does *Etapy*, although it also differs from it in interesting ways.

The second book was edited by Giuseppe Cossu, who translated *The Making of Mind* (Luria [Lurija], 1987), and was republished in 2023, this time with no indication of its American source. However, Luria had originally asked Cossu to translate the entire typescript of *Looking Back* in February of 1976 (Cossu, 1987, p. 1) and wrote to Luciano Mecacci on May 27: “My book is ready, and Giuseppe is translating it into Italian.”^7^ However, for both professional and private reasons Cossu only translated the first 46 pages before leaving Moscow. At that point, he left these pages behind but brought the rest of the typescript and his translation to Italy.^8^

It is recommended to mention all sources so that the complexity of the bibliographical situation be graspable, though it may be tedious to read through the list. However, it is important to be aware of the said complexity for at least one reason: how people extensively used (or simply referred to) which sources is a relevant aspect of the history of Lurian life-writing.

Only *two brief texts* that were written by Luria himself, one chapter and one article, were published during his lifetime. As for the so-called book-length autobiography, all twelve versions, translations and reprints, with or without supplementary material, in seven languages, were published posthumously.

We must emphasise that some versions are more or less faithful translations of the March 1976 typescript *Looking Back* (Luria, 1979a; Luria [Lurija], 1983), others are translations of *The Making of Mind* (Luria, 1979b [second edition with substantial additional material 2006]; Luria [Lurija], 1987 [second edition 2023]; Luria [Lurija], 1992 [second edition 2017]), and still other books (Luria, 1985; Luria [Lurija], 1993) rely on the Russian publication *Etapy* (Luria [Lurija], 1982 [second edition 2001]) – which as we have already mentioned turns out to be basically a translation of *The Making of Mind*. All this published material, along with some additional typescripts we have found, constitutes Luria’s complete genuinely autobiographic or else autobiographic-and-heterobiographic *life-writing* (see Table 1).

**Table 1:** A. R. Luria’s published and unpublished life-writing

Luria, A. R. (1973). The long road of a Soviet psychologist. *International Social Science Journal*, *XXV* (1/2), 71–87. In French in the same journal: Regards d’un psychologue soviétique sur le chemin percouru (pp. 74–91). An Italian translation was published in a collected volume containing selected writings of Luria on neuropsychology and neurolinguistics in 1974: Luria [Lurija], A. R. (1974). Fasi di una ricerca (appunti di uno psicologo). In A. R. Luria [Lurija], *Neuropsicologia e neurolinguistica* (Eds.: E. Bisiach & L. Mecacci, pp. 3–21). Rome: Editori Riuniti. Luria, A. R. (1974). A. R. Luria. In Gardner Lindzey (Ed.), *A history of psychology in autobiography, volume VI* (pp. 253–293; translation by M. Cole of a typescript from 1964). Englewood Cliffs, New Jersey: Prentice Hall. Luria, A. R. (1976a). *Guardando indietro*. *Moscow 1976* (translation by Giuseppe Cossu of a part of *Looking back* into Italian). Private Archive Giuseppe Cossu (Padua). Luria, A. R. (1976b). *Looking back. The life of a psychologist in retrospect*. *Moscow March 1976* (with handwritten corrections by Linda Wertsch; the earliest version of the typescript available to us; however, it is incomplete as the first 46 pages were left in Moscow and only exist in an Italian translation [see above]). Private Archive Giuseppe Cossu (Padua). Luria, A. R. (1976c). *Looking back. The life of a psychologist in retrospect*. *Moscow March 1976*. Jerome Bruner Personal Archive. 2017.183 Box 2. Harvard University Archives. Luria, A. R. (1977). *Looking back. The life of a psychologist in retrospect*. *Moscow March 1977*. Private Archive Michael Cole (San Diego). Luria, A. R. (1979a). *Mirando hacia atrás. Obra postuma. La vida de un psicólogo soviético en retrospección. Moscú 1976*. (Translation by M. Pérez Pàmies and J. Peña Casanova; preface by L. Barraquer-Bordas). Madrid: Ediciones Norma. Luria, A. R. (1979b). *The making of mind. A personal account of Soviet psychology*. (Eds.: M. Cole and S. Cole; introduction and epilogue by M. Cole). Cambridge, MA.: Harvard University Press. Lurija, A. R. (1979c). Una scienza romantica. Rittrati non immaginari (translation by G. Cossu of Romantic science. Unimagined portraits; unpublished typescript Moscow 1976). In A. R. Luria [Lurija], *Viaggio nella mente di un uomo che non dimenticava nulla. In appendice due scritti inediti* (pp. 108–116). Rome: Armando Armando. Luria [Lurija], A. R. (1982). *Etapy proedennogo puti. Nauchnaya avtobiographiya*. (Ed.: E. D. Homskaya; preface by E. D. Homskaya; 2^nd^ edition: 2001). Moscow: Izd-vo. Mosk. Univ-ta. Luria [Lurija], A. R. (1983). *Uno sguardo sul passato. Considerazioni retrospettive sulla vita di uno psicologo sovietico*. (Ed.: M. S. Veggetti; introduction to the Italian reader by M. S. Veggetti; translated from English by G. Noferi, and M. S. Veggetti from Russian; original title: Etapy proedennogo puti. Iz zapisok sovetskogo psihologa [sic]; Looking back. The life of a psychologist in retrospect; copyright: VAAP, Moscow 1976). Florence: Giunti Barbèra. Luria, A. R. (1985). *Itinéraires d’un psychologue*. (Ed. by E. D. Homskaya; preface by E. D. Homskaya; translation from Russian by G. Dupond in collaboration with G. Molinier). Moscow: Éditions du Progrès [=Progress]. Luria [Lurija], A. R. (1987). *Il farsi della mente. Autobiografia di A. R. Lurija*. (Eds.: M. and S. Cole; introduction and epilogue by M. Cole; preface and translation by G. Cossu; translation of *The making of mind*; 2^nd^ edition 2023 by the same publisher but without any information as to the edition by M. and S. Cole). Rome: Armando Armando. Luria, A. R. (1992). *A construção da mente*. (Translation from English by M. Brandão Cipolla; no information as to the edition of M. and S. Cole; 2^nd^ edition 2017). São Paulo: Ícone. Luria [Lurija], A. R. (1993). *Romantische Wissenschaft. Forschungen im Grenzbereich von Seele und Gehirn*. (Translation from Russian by A. Métraux; with a text by O. Sacks.) Reinbek: Rowohlt. Cole, M., Levitin, K. & Luria, A. (2006). *The autobiography of Alexander Luria. A dialogue with The making of mind* (including a DVD). New Jersey: Lawrence Erlbaum.	

### Comparative Analyses

Luria’s life-writing is anything but homogeneous, as should be clear by now. Our comparative analysis of the material available to us aims to articulate overarching similarities and specific differences between relevant documents. While concentrating on textual features, we do not discard apparent minutiae, such as titles.

The two self-portraits published during Luria’s lifetime display him (or at least were intended to display him) not as a private person working in a socially and legally defined context of research in labs, clinics, and hospitals and thus defined by his professional duties and their aims, but rather as a scholar whose activities as husband, father, friend, and more-or-less appreciated colleague are deemed to be void of autobiographic significance. The scientific achievements themselves, and more specifically that they were those of a *Soviet scholar*, were to be told to a worldwide audience that Luria assumed was not particularly well informed concerning Soviet psychological research (which he would have in any case been able to tell from Boring’s letters, see above).

The so-called autobiography by and of Luria turns out to be closely related in intention to the early sketches for self-portraits. However, there are some obvious differences that we ascribe to the editors who reworked and either shortened or expanded various parts of the original version of *Looking Back* using material from other written sources or personal communications with Luria himself.

Once again, *Mirando hacia atrás* (1979a) is a Spanish translation of the 1976 typescript of *Looking Back*^9^ that differs from *The Making of Mind* (1979b) in length and content. *Mirando* is shorter and more focused on neuropsychology and neurolinguistics, whereas *The Making of Mind* contains more material on Luria’ contributions to cultural psychology. And yet, the different account of the latter version was fully supported by Luria who had repeatedly agreed with the editorial re-working intentions laid open by Cole in conversation and by other means of communication (see below).

The Spanish version was badly proofread, if at all, and it reproduced the errors in the original text and even added new ones. For example, Luria’s typescript quotes the beginning of Freud’s letter to the author as “Sehr geehrter Herr President” (instead of “Präsident” [*Looking Back*, 1976c, p. 6]), as does the Spanish edition (*Mirando hacia atrás*, p. 13). He refers to his formative years as “Schuhljahre” (the correct spelling is “Schuljahre”) (p. 108); so does *Mirando* (p. 84). Similarly, Kurt Goldstein’s “Die Lokalisation in der Großhirnrinde” is misspelled as “Die Lokalization in der Crosshirnrinde” (*Looking Back* 1976c, p. 111), which *Mirando* (p. 86) does not correct. All these errors, and other similar ones, could have been avoided by looking up the titles in readily available bibliographies and catalogues.

Moreover, *Looking Back* (p. 223) refers to the Argentinian author Jorge Luis Borges’ story *Funes el memorioso* (*Funes the memorious*, Borges [2007/1942]) as “the well-known short novel by J. Borges ‘Funes the Memorial’”, which *Mirando* (p. 160) blindly und unhesitatingly renders literally as “la conocida novela corta de J. Borges *Funes the memorial*.” Further errors include dating Vygotsky’s move from Gomel to Moscow to the fall of 1925 instead of 1924 (*Mirando*, p. 27) and not checking the identities of persons mentioned in the text they were translating: thus, Liya Solomonovna Geshelina and Bljuma Wulfowna Zeigarnik are referred to as “el doctor B. Zeigarnik” (p. 35) and “el doctor L. Geshelina“ (p. 34) instead of with the feminine form of the title, “la doctora”. Finally, in the chapter on romantic science of the 1976 typescript, the term “unimagined portraits” (p. 210) was misleadingly transformed as “retratos inimaginables” (*Mirando*, p. 153); however, the word “inimaginable” does not mean “unimagined” but rather something like “unimaginable” or “beyond imagination”, which is clearly not what Luria intended to convey.

We have compared the typescript of 1976 to both that of 1977 and the Coles’ version of *The Making of Mind* published by Harvard University Press and found that the 1977 typescript constitutes an intermediate state between the other two. However, it still resembles the earlier typescript much more than the published monograph *The Making of Mind*, even though the latter was elaborated from it.

What are the main differences between *Looking Back* and *The Making of Mind*? Let us first look at the table of contents. Unlike the Spanish edition and both typescripts, the Coles’ version as materialised in *The Making of Mind* lacks the foreword by Luria and consists of only ten chapters rather than twelve.^10^

While *The Making of Mind* contains some limited information on Luria’s family, in particular his father (p. 18 and pp. 24–25), the 1976 typescript entirely omits the topic of father-son-bonds: indeed, Luria declares (p. I) that “[t]he reader will find here neither autobiography, nor the pedegree [sic] of the author’s family, nor the influence of his father, no Oedipus complexex [sic].” The expression “neither autobiography” ought to be taken to declare that memories centred on the author’s so-to-speak private person are irrelevant and that a *scientific* autobiography is essentially a record of *enduring ideas*, not individual persons who come and go. This core idea is prominently advanced at both the beginning and at the end of the 1976 typescript, with slightly different wording: “People come and go. Ideas, deeds and events remain” (p. II) and “People come and go. But the creative sources of the great historical events – and the important ideas and deeds – remain” (p. 224). Thus, Luria intended his autobiography to be a portrait of a participant in the Soviet enterprise of psychology, in which the author made himself visible as merely one among innumerable scientists acting within an overarching history. Luria presents himself repeatedly as an “average” or indeed “modest scholar” (p. 2, and p. 224, respectively), self-descriptions that lack any exceptional meaning, except that they span such a long period in time (p. I).

Compared to the 1976 typescript, *The Making of Mind* includes a wealth of information on the post-revolutionary atmosphere at the University of Kazan, on Luria’s early reading (Windelband, Rickert, Dilthey, Höffding, and others), and on his strong interest in psychoanalysis, to which he turned not least to solve the crisis of a psychology torn between nomothetic and idiographic approaches: that is, between explanation and understanding.

This interest in psychoanalysis is described in various ways. On the one hand, we encounter the narrative of Luria’s foundation of the psychoanalytic society at Kazan; on the other, works by Sigmund Freud, Alfred Adler, and Carl Gustav Jung are mentioned as objects studied and attentively received. In addition, Luria mentions a project of his own, called “experimental psychoanalysis” (cf. *The Making of Mind*, p. 32; the latter term occurred already in the 1976 typescript, p. 21). Later, experimental psychoanalysis happened to be linked to the monograph on *The Nature of Human Conflicts* (Luria, 1960/1932). However, the 1976 typescript – in contrast to *The Making of Mind* – keeps the complex of psychoanalysis at its narrative margins. Hence, while autonomously writing his autobiography Luria presents his early attraction to psychoanalysis (along with some other more or less scientific subjects) as a kind of immature play with no further importance: “This pre-history of my work in psychology has nothing to do either with the further development of the Soviet psychology, nor with my own further work in science” (*Looking Back* 1976c, pp. 6–7). Compare this passage with the “pre-history”-passage in *The Making of Mind* (p. 27): “Throughout this period of my life I was naively groping. Still, after fifty years, I have the feeling that many of these activities were significant in my further development as a psychologist. In later years the surface appearance of my research changed a great deal. But the central themes that had guided my initial efforts remained.”

Let us focus on another aspect of Luria’s self-description. Though *The Nature of Human Conflicts* receives little attention in the 1976 typescript (pp. 19–20), *The Making of Mind* highlights its relevance due to the editors’ addition of examples taken from that monograph (which, as we may recall, was originally published in English). These examples cover topics ranging from the study Luria conducted with Leontiev on conflicts in students awaiting examination (political or academic) to his study on convicted criminals (*The Making of Mind*, pp. 32–36; see also Luria, 1960/1932, pp. 47–127).

The two expeditions Luria led to Central Asia are also briefly summarised in the 1976 typescript (colour perception, the application of syllogistic rules by subjects; cf. pp. 51–57). However, *The Making of Mind* contains a series of examples taken from the empirical material gathered during these expeditions to Uzbekistan and Kirghizia. Page after page describes vivid examples whose subjects become embodied persons, such as Rakmat, introduced by name and described as “a thirty-year-old illiterate” whose categorical thinking they analysed in the field (pp. 69–71).^11^

We should here add a remark on the history of these expeditions to Central Asia. The field studies carried out in Uzbekistan and Kirghizia came under political attack in the 1930’s (van der Veer & Valsiner, 1991, pp. 253–255) – one of the reasons Luria reoriented his professional activities – and their results were published in detail only decades later. The 1976 typescript (p. 51), however, informs the reader that “[i]t was not until 40 years later that I was able to publish a short summary of our results. My other research … prevented immediate publication.”

In other words, *Looking Back* plays some things down. At one point (p. 60), Luria asserts after relating the expeditions and other research he carried out before 1935 that “I have finished my digression from the main thrust of my life in science.”

The differences between *Looking Back* and *The Making of Mind* may receive differing interpretations. On the one hand, one might assert that the author of *Looking Back* was intending to highlight his *neuro*scientific contributions to Soviet research. On the other hand, one may be inclined to recognize that the author did not mind presenting himself as a successful Soviet cultural psychologist.^12^ Rather than give preference to either interpretation, it seems more appropriate to accept both as plausible, since autobiographies, written down over months, if not years, do not consist of recollections put together according an unchanging “recollection software.”

## What about Romantic Science?

Luria’s case studies on Shereshevsky and Zassetsky epitomise – or, rather, have often been said to epitomise – Luria’s romantic science. Taken together, *The Mind of a Mnemonist* (Luria, 1987/1968) and *The Man With a Shattered World* (Luria, 1975/1971) may also be understood to distinguish themselves from the self-contained scientific discourse of the neuropsychologist and neurolinguist Alexandr Romanovich Luria (see Hawkins [1986] on Luria’s “art of clinical biography”). In other words, the typescript of 1976 (p. 220) implies that the perspective or style (to speak vaguely) of the two case studies makes them paradigmatic instances of “the approach of ‘romantic science’”. Some pages earlier, Luria’s text states that he had “tried to revive the tradition of a ‘romantic science’” (p. 217) when writing these case studies. But no information is offered which tradition Luria had in mind, or even whether he was using the expression of “romantic science” to refer to any recognisable tradition of doing science. Which is to say that we have to accept both Luria’s terminological choice and the manner in which he instantiated in writing whatever he had in mind when using the term “romantic science.” One may refuse to overstate the relevance of “romantic science” for Luria’s work, as Oliver Sacks and some of his followers have done. However, this is not a valid reason for minimizing the role Luria gave to “romantic science” in his life-writing, since clinical case studies (or histories) are to be found as well in several of his “classical” monographs.

The 1976 typescript (p. 217) alludes briefly and in hindsight to the aim of Luria’s idea of romantic science: “In each book [i.e. the two case studies made public in book form] I dealt only with *one man*, trying to approach the ‘individual laws’ of his mental life. It was a new attempt to resolve the conflict between the ‘Nomothetic’ and ‘Idiographic’ sciences, which attracted my attention more than 50 years ago. Couldn’t I follow the steps of Walter Pater, – who wrote, ‘Imaginary Portraits’ (1887), by trying to describe ‘Unimaginary Portraits’?” But this, Luria’s text immediately warns, “would be an awesome task. It is almost impossible to write an analytical description of a human being taken at random from a crowd, singling out his decisive personality trait and indiscerning [sic] its ramifications. That is why I chose a man who had only one, but unquestionably decisive feature that made him different from all ogher [sic] men, that played a decisive role in determining his unique character and personality.” This man turns out to have been the mnemonist Shereshevski, a person endowed with a limitless memory, who unable to forget (to make things easier than they were in unimaginary reality), also increasingly became unable to manage everyday life.

The other man, Zassetsky, was a similarly unique person who succeeded in a nearly super-human way at restoring his personality out of nothing after a massive brain injury received during combat.

The reference to Pater in the 1976 typescript is, however, at best ambiguous. Whereas Pater’s portraits addressed the issue of *how* to convey the image of a fully realised person by writing, Luria’s unimaginary portraits were presented as *detailed*,* lively descriptions* of very unusual and probably even unique human beings and thus far from being, somehow, *experiments in how* to vividly portray human beings. In other words, while Pater’s portraits depicted imagined human beings whose dispositions and mental properties were far from unique, turning these portraits into achievements of artistic writing in prose, Luria’s portraits depict extraordinary cases of human existence in ordinary language.

Another point concerning “romantic science” also needs to be touched on. Chapter 12 of the 1976 typescript (p. 210) is called “Romantic science: Unimagined Portraits.” The chapter opens with these words: “At the beginning of this century, one of the wellknown German scholars (was it Max VeRworN? ) assumed that men of science can be divided into two large classes, classic and romantic.” (The upper-case characters “R” and “N” are overwritten, probably in Luria’s handwriting.)^13^ Yet, it was not Max Verworn who classified scientists as either romantic or as classic but Wilhelm Ostwald, who suggested that scientists be typified according to the pace of their research activity. However, the 1977 typescript sent to Michael Cole correctly named Ostwald as the early twentieth-century German scholar in question (see also below).

Romantic scientists, according to Ostwald, dealt rapidly with research problems, turned their attention to more than one key object of investigation, and did not hesitate before making their results public. Classical scientists were highly thoughtful, reached public visibility slowly, and stuck to a single key object of investigation to get to know it as thoroughly as possible (Ostwald, 1909, p. 371 sqq.).

There is no doubt that Luria hesitated when composing *Looking Back*, a narrative whose very title was appropriate for establishing a link to this dichotomy of romantic and classical science. Yet in the end, the self-portrait published as *The Making of Mind* (as well as its derivative editions and translations) mistook Verworn for Ostwald and substituted a literary genre supposedly conceived of by Verworn in place of the Ostwaldian distinction based on insights drawn from some vague personality theory.

Both the 1976 typescript and *The Making of Mind* describe Lurian “romantic science” fairly extensively. Even taking into account the different topics emphasised by the two versions, they give different levels of prominence to romantic science. Approximately half of the typescript of *Looking Back* is devoted to neuropsychological and neurolinguistic topics. This is clearly more than in *The Making of Mind*, in which Michael and Sheila Cole present Luria not only as a pioneer of neuropsychology and neurolinguistics but also a champion of cultural psychology, a recognised developmental psychologist, and as someone who favoured romantic science to an unprecedented degree. Even more importantly, in the chapter on romantic science in *The Making of Mind* the editors reworked the typescript they had received from Luria to play up the life and work of its “hero” and show that romantic science had been an important issue for the young scholar in the earliest years of his career. During that period, Luria was attempting to bridge the idiographic and nomothetic psychologies whose divergence was causing what he diagnosed as a crisis of psychology. *The Making of Mind* thus returns on a meta-level to the point where it began, while by the end of the 1976 typescript these steps taken by the young Luria have lost most of their relevance and no longer played any role for the mature Luria: his contributions would live on, but not he himself as a mere modest scientist.

In short, we maintain that in the typescript of *Looking Back* Luria predominantly presents himself in the guise of a neuropsychologist and neurolinguist and emphasises his scientific endeavours (and contributions to the progress of Soviet research) while often leaving the significance of his other research activities in the background. This typescript describes his academic life as an ascending pathway, even though his career followed anything but a straight line.

However, the Luria of *The Making of Mind* portrays himself as a cultural and a developmental psychologist, a romantic scientist, a researcher who in a way achieves a resolution to the crisis of psychology, at least within his own work – *as well as* an innovating neuropsychologist and neurolinguist. The history of various research programmes is presented more vividly than in the typescript. Further, he appears as the humble disciple of the great genius Vygotsky, without the typescript’s often rehearsed claim that he himself is just an average scientist. In *The Making of Mind*, the key person of the story appears above all in his role as an active scientist; yet at least a few bits and pieces of his life beyond science and of the social atmosphere in which he lived can also be sensed. And, as indicated above, the linear construction of Luria’s (academic) biography in the typescript of *Looking Back* becomes a circular one in *The Making of Mind* that resonates with Michael Cole’s obituaries of Luria, first in the *American Psychologist* (Cole, 1977) and a year later in a modified version in *The American Journal of Psychology* (Cole, 1978).

## Whose Autobiography?

Philippe Lejeune defines the concept of autobiography as a “retrospective narrative in prose by a person on his/her own existence, thereby putting the emphasis upon his/her individual life, and in particular upon the history of his/her personality.” (Lejeune, 1996, p. 14^14^) To be rightly called an “autobiography”, according to Lejeune, a text must meet the following criteria: it must be a narrative *in prose*, it must deal with the history of an individual person, the author must be identical with the narrator (whose name must hence denote an existing person), and the narrator must be identical with the person narrating himself/herself in retrospect (Lejeune ibid.).^15^

We assume, here, that a translation of Luria’s original typescript should be considered equivalent to the self-narrative written in English, whatever the reason why he did not write his autobiography in his native tongue. *Looking Back* was obviously written by Luria, who obviously narrated his own life in English, signed the typescript with his own name and thereby authenticated the narrative as truly being the history of his life.

As asserted above, Luria’s two sketches or fragments of truly autobiographic scope fulfil the criteria established by Lejeune. So does *Looking Back*. However, since the original typescript of *Looking Back* has never been published in the language its author chose to write in, the only authentic, fully realised (i.e. more than fragmentary) autobiographic account of Luria’s life available in print is the Spanish translation of the 1976 typescript. It, and only it, counts as Luria’s autobiography based on Lejeune’s criteria that we have adopted here.

This implies that we do not regard *The Making of Mind* as original, authentic biographic narrative. But it is also obviously neither a text of semi-fiction, nor a biography of Luria whose author happens not to be identical with the narrator (that is, the “I” of the text). If it is neither an authentic autobiography nor the opposite of an authentic autobiography, what is it? In one word: it is an autobiography with heterobiographic *addenda*, a blend of autobiographic parts and heterobiographic bits by at least two, and (depending on the circumstances of its publication) possibly even more authors, who by logical necessity cannot be identical with the person (Luria) narrating his own life.

Let us first consider some purely linguistic details of the 1976 typescript.

There is no doubt that Luria narrated what he had lived through in a text that he typed while recovering from a heart attack. In a passage of it about Vygotsky, he wrote: “It was a time Vygotsky was very interested in patients with parkinsonism – that time a newly described disease. The pathological state of the subcortical motor ganglia disturbed the involuntary flow of the movements so deeply, that after a very short time – less than one minute – movement became impossible, and tremor or pathological rise of the muscle tone blocked their further execution.” (Luria, 1976b, p. 69).

**Fig. 1:**
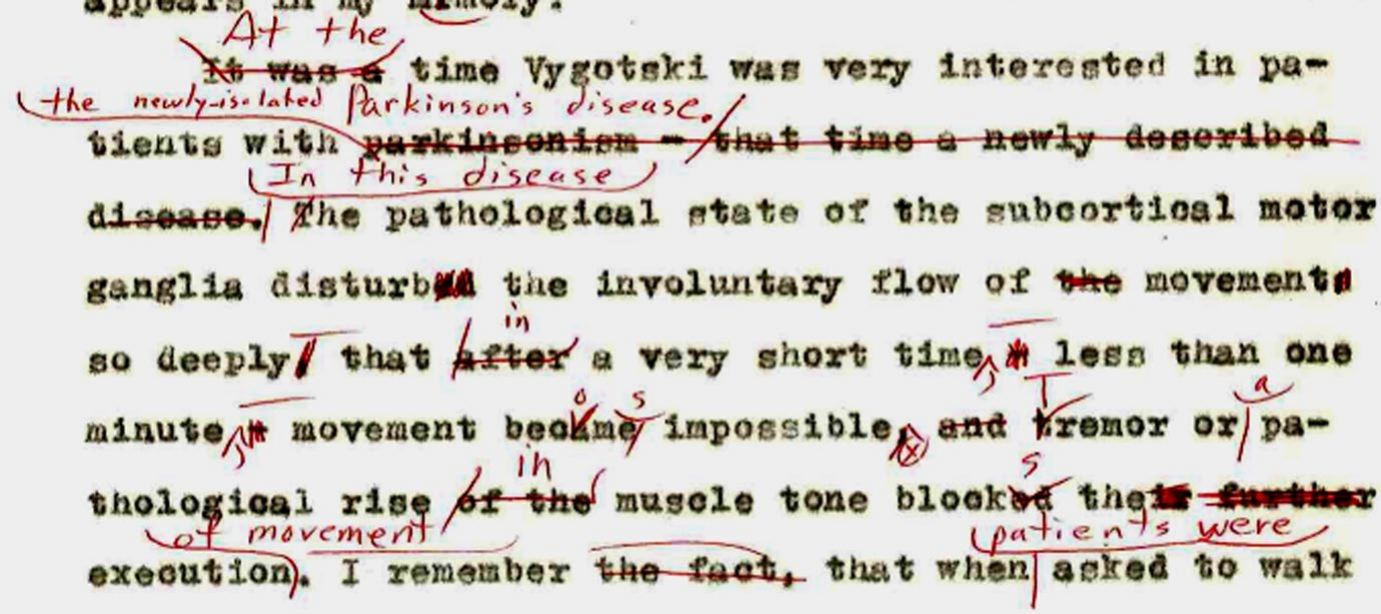
Fragment from page 69 of the typescript *Looking Back* (1976b) with handwritten corrections by Linda Wertsch (courtesy Giuseppe Cossu)

This passage, as well as the typed text up to page 139, was revised by a native English speaker, Linda Wertsch, a journalist who was then married to James Wertsch. The revised passage reads: “At the time Vygotski was very interested in patients with the newly isolated Parkinson’s disease. In this disease the pathological state of the subcortical motor ganglia disturb the involuntary flow of movement so deeply that in a very short time, less than one minute, movement becomes impossible. Tremor or a pathological rise in muscle tone blocks the execution of movement.” (ibid.; see also Fig. 1 above).

How should we characterise the result of the editing undertaken by someone whose identity does not correspond to that of the autobiographic author?

Consider for a moment the original French version of Jean-Jacques Rousseau’s *Confessions*, of which the first part was published in 1782 and the second in 1789. Several translations of this autobiography into English, Italian, German, and other languages have appeared over time. The literal non-identity between the original and its translations does not modify the status of this autobiographic work. A very narrow interpretation of Lejeune’s criteria would disqualify the translations of Rousseau’s *Confessions* from being considered his autobiography since the translators are not identical with the author of the original text. For good theoretical reasons, and even more so for practical reasons, it should be granted that the proto-version of the 1976 autobiographic text (Luria, 1976a, 1976b) and its revised version (Luria, 1976c) both authentically embody Luria’s autobiography, even though the text of the 1976 typescript (1976c) testifies extensively to the English native speaker’s revision.

Now, the text of the proto-version (from p. 139 through p. 250) shows no trace of any ‘polishing’ revision. But the ‘clean’ 1976 typescript also differs from its proto-version in the same way as its beginning (from p. 1 through p. 138) from its antecedent version. This means that some unknown person *did* revise the remaining parts of the proto-version not revised by the native English speaker.

For the sake of illustration, we quote a brief passage (a) from the non-revised part of the proto-version and (b) from the 1976 typescript after having undergone revision by someone unknown to us and to all those whom we contacted in search of expert information.

- “At the beginning of October 1941 I went to Cheliabinsk and then to a small village Kissegach and was appointed as a Scientific adviser of a large hospital with ca 400 beds. Laboratories and training rooms were established, a team of my Moscow collaborators was transported to this place, and in a month the Hospital began its work.” (Luria, 1976b, p. 141).
- “In the beginning of October, 1941, I went to Cheliabinsk and then to a small village, Kissegach, where I was appointed a scientific adviser for a hospital with 400 beds. Laboratories and training rooms were established. A team of my Moscow collaborators was brought in. And within a month the hospital had begun its work.” (Luria, 1976c, p. 127).

Fine-grained textual analyses show that the ‘clean’ 1976 typescript was used by the Spanish translators for the publication of *Miranda hacia atrás*.

But the text of this ‘clean’ 1976 typescript was revised one more time by Luria. We assume that this further revision was Luria’s and that he completed it on his own (except for the technical making of the final typescript as such).

Before recounting how *Looking Back* in its final version as the 1977 typescript reached Michael and Sheila Cole, who made it into the famous monograph *The Making of Mind*, we quote a passage from the 1976 typescript (Luria, 1976c), followed by the corresponding passage from the later typescript:

- “We even had some basic concepts concerning the syndromes of local brain lesions, and, as vague these concepts were, we could start with them and improve them in our day = to = day work. Our work in the Urals continued for three years and was transferred to Moscow after the end of the war. The facility in the Urals reverted to a sanatorium.” (Luria, 1976c, p. 128).
- “We even had some basic concepts concerning the syndromes of local brain lesions, and, as vague these concepts were, we could start with them and improve them in our day = to = day work. Some Neuro-surgical apparatuses, technical devices including that needed for EEG studies were brought from Moscow, the town, which was under permanent bombing; a histological laboratory was established.” (Luria, 1977, p. 163).

Though the typescripts differ in length as well as in the details narrated, both are still genuine authentic autobiographies, despite the amount of external linguistic input that (rather slightly) transformed what Luria had written or typed. Since the 1977 typescript has also never been published, the sole publicly available authentic self-portrait in prose by Luria is the Spanish translation of the 1976 typescript.

The events leading to, and following, Michael Cole’s receipt of the 1977 transcript of *Looking Back* are decisive in reaching a well-founded assessment of the heterobiographic character of *The Making of Mind*, which is conventionally regarded as Luria’s autobiography. What kind of heterobiographic elements does one encounter when comparing the untouched 1977 typescript with *The Making of Mind*? One typical example is likely to illustrate the extent of modifications that mark Luria’s published life-writing as edited by the Coles.

A passage of the 1977 typescript which is identical to the corrected passage of the 1976 typescript (see Fig. 1) reads thus:

“At this time Vygotski was very interested in patients with the newly-isolated Parkinson’s disease. In this disease the pathological state of the subcortical motor ganglia disturb the involuntary flow of movement so deeply that in a very short time, less than one minute, movement becomes impossible. Tremor or a pathological rise in muscle tone blocks the execution of movement.” (Luria, 1977, p. 87).

The corresponding passage quoted from *The Making of Mind* reads:

“We were more successful when we began to observe patients suffering from Parkinson’s disease. Parkinson’s disease affects the subcortical motor ganglia so that the flow of involuntary movements is disturbed. We observed that tremors occurred shortly after patients suffering from this disease started to carry out an action.” (Luria, 1979b, p. 128).

A detailed comparison of the two versions suggests that the text of *The Making of Mind* is not an authentic autobiography, as it does not fulfil a key criterion established by Lejeune. Indeed, the second passage differs from the first in both content (the name of Vygotsky has been deleted, Parkinson’s disease is no longer said to be newly isolated, etc.) and with respect to the linguistic outlook (syntax, details of style).

Which is to say that we face a paradox. On the one hand, things appear to be uncontroversial, because a genuine autobiography of Luria exists in published form as the Spanish translation of the 1976 authentic autobiographic typescript. On the other hand, there is also a portrait of Luria’s scientific life that resulted from some co-writing by another hand to supplement Luria’s narrative. Depending on the viewpoint judged adequate, things are not really so clear after all, so we ought to determine who contributed which elements to the final published version of *The Making of Mind*. To do so, we must briefly look at the intricate history of the published book.

## On the Making of ***The Making of Mind***

Towards the end of 1975 and early 1976, Luria began to work on a text that then turned into his autobiography. On March 2, 1976, he wrote to Michael Cole on the work in progress: “It will be a Retrospect of my whole life in science, half scientific, half lyric. The title will perhaps be ‘*THE LAST BOOK*’ with a subtitle ‘A Life of a Psychologist in Retrospect’. I am writing the book directly in English.” He then added: “I shall try to send you a preliminary copy not for publication, but for a [sic] preliminary impressions.”^16^

The finished text was typed (we do not know by whom) and more or less carefully read by Luria, who corrected typographical and other errors. The resulting typescript bears the title *Looking Back* and the date “March 1976”, though this does not imply that the typescript itself was completed in that month, since this date may be when the longhand manuscript was completed.

The subsequent events overlapped. We will try to present them in an order that will make the narrative as clear as possible.

On March 29, Luria suffered a severe heart attack. He wrote about this to Jerome Bruner on May 26, 1976 and also informed him that “[d]uring the month of my illness I typed my English manuscript of the new book …‘*Looking Back*’ with a subtitle ‘an autobiographic novel’, where I put together all my life in science from the first years to the end. It is written in a free style, and now, when my English is revised and edited by the wife of my friend Jim Wertsch – the book is ready.” We gather from these statements that Luria typed his manuscript in March or April and gave it to James Wertsch’s then-wife Linda, a journalist and native speaker to improve his imperfect English. Linda Wertsch’s work on the typescript ended on page 138.

However, since he gave this text with Wertsch’s handwritten corrections (see Fig. 1 for an example), along with the rest of the very first typescript, to Giuseppe Cossu who had agreed to translate *Looking Back* into Italian (which he started to do while staying at Moscow for his own neurological research), we must assume that Luria was *at that time* convinced this was the final version of his self-portrait and ready for publication in English: after all, he considered the very same text good enough to translate into Italian and publish. As he wrote to Luciano Mecacci on May 27 – the day after having writing to Bruner, “[m]y book ‘Looking Back’ is ready, and Giuseppe is translating it to Italian.”

Around the same time *Looking Back* was officially sent to the Paris-based French publisher Masson. However, they rejected it and sent it to Barcelona, where the Spanish translation was expected to be done. Meanwhile, the same text was sent to Michael Cole in New York, although we do not know by what route. In any case, some weeks after the receipt of the ‘clean’ 1976 typescript (that edited by Linda Wertsch), Cole conveyed his first impressions after reading the text to Luria in a letter, as we infer from the wording of the latter’s response to his friend Cole on September 15, 1976: “… ad ‘*Looking Back*’. I fully agree with all your remarks. You are right: the paper is something between a narrow autobiographic novel and an attempt to write a small part of Soviet psychology. … I fully understand that it hardly will be good for print. That is why my proposal is: please do *postpone its publication*, and do *not* make any steps to contact with a publisher.” In another letter, on October 16, 1976, Luria wrote to Cole (probably in response to Cole’s October 6 response to the previous letter sent by Luria telling him that he would need some time to seriously assess the text of the autobiography): “I shall wait very quietly to the development of things with the ‘Looking Back’; please, don’t hurry; the Ms is *in your hands* and all your comments will be carefully considered. *Of course*,* that was only a* *preliminary* *text*,* which has to be carefully elaborated*” (our emphasis). Which is to say that Luria had changed his mind after having received Cole’s remarks on a typescript that had already been sent to Paris and Barcelona as being a (previous) final version ready for translation (for which errors in English grammar, syntax, and idiomacy would not be noticed anyhow). In other words, it is very remarkable that Luria had *in good faith* considered the 1976 typescript good for publication, but that after considering Cole’s comments, he seems to have been convinced that he would better change his mind. Are we therefore compelled to consider Luria’s rejection of the *sole* authentic autobiographic version (i.e. the Spanish translation) as a mistake? And did he *inadvertently* handle matters of his self-portrait, since in the October 16 letter he stated that the real, the final, the authentic autobiography would have to be carefully elaborated *later on*? The answer depends, once again, on the concept of autobiography – as well as upon the interpretation of the inferred motives of the author: was he, in the end, keen to promote himself through a self-portrait in English prose, or did he weigh arguments with which Cole (and possibly some other persons) confronted him?

According to Lejeune’s approach, autobiographies refer to not one but two subjects at the same time. These texts deal with (describe, explain, account for, etc.) on the one hand the narrator’s past (or parts of his/her past), and on the other the narrator when (and while) writing on him/herself. Hence, an autobiography’s subject is two-fold – it is the “I” that lived once upon a time at some place (or at several places in a row), who did this and that; and it is also the “I” who is writing down (or has written down earlier in the text being written, or who writes that he/she intends to write later on) his/her narrative of his/her life (or parts thereof). Thus, Luria states in the *Preface* of the 1977 typescript of *Looking Back* (Luria, 1977, p. I) that “This is a rather personal book. The reader will find here neither autobiography, nor the pedegree [sic] of the author’s family, nor the influence of his father. … Rather, the author will find here a collage of what the author seems most important [sic] in his long life.” This leaves no doubt that the subject of this passage, “a rather personal book”, is the object of the author’s explanation of what his book is and is not; hence the author speaks of (and especially for) himself at the precise moment at which he writes as the author of his autobiography. But when narrating what he did at which place and time in the company of other persons, the same author does not speak (write) for himself as the author of his autobiography, but for himself as the actor who he was in the circumstances he remembers when writing his text. The crucial point in all this is that the moment of recollection differs from the recollected (memorised, mnestically awakened) moment, whereby there exists no necessary connection between the wording chosen at the first moment and the recollected event(s) described at that moment. This entails that the author may (like Luria) change the wording and even the specific content of past lived events without affecting the authentic character (or nature) of his telling the readers what his past life had been. This means that neither revising a proto-version of his autobiography, nor revising the first revision of the proto-version alters the authenticity of his autobiography as set down in the typescripts of 1976 and 1977, respectively.^17^ Non-identity in wording does *not* result in non-identity of the genuinely autobiographic narratives.

We assume that Luria again revised the text on which Cole had made more or less serious or far-reaching comments. It was clear to Luria by the end of 1976 that he had not yet achieved a text that was ready for publication. It was also clear in March 1977 that Sheila and Michale Cole would edit Luria’s text to make it acceptable to a publisher in the United States or another English-speaking country. The preface of the 1977 typescript (p. II) states: “It would be unfair if I should not express my deepest thanks to my dear friends – Michael and Sheila Cole. Their suggestions as well as their editorial work were really invaluable, and if this book appears – it is only the result of their friendly help and our joint work.”

One must not infer from the reference to the editors’ “suggestions as well as editorial work” (of which Luria asserts that they “were really invaluable”) that Sheila and Michael Cole had already completed their work in March 1977. Luria formulated the sentence as a statement that would appear in the publication and thus at future point in time at which the suggestions he had received earlier and the editorial work done by the editors would belong to the past.

In any case Michael Cole and Luria worked together on the typescript in April 1977. On May 26, 1977, Luria wrote to Bruner that “I spent April in cooperation with Mike Cole on my ‘Looking Back’”. The finished 1977 typescript that was sent to Cole must be taken as another version (in terms of wording, content, etc.) of the same authentic autobiography *qua* narrative of an “I” who appears as “I” having acted in such and such manner in the past, and insofar as this “I” is remembered and given embodiment in prose.

At any rate, the director of the Export & Import Department of the Copyright Agency, Yuri Gradov, wrote a letter to Cole on April 29, 1977, stating: “On Prof. A. R. Luria’s request we are sending you attached to this letter a revised copy of his manuscript ‘Looking Back’. We hope to hear from you soon about the progress in finding a publisher for this new work by Prof. A. R. Luria.”^18^

On June 8, 1977, Luria wrote to Cole that “[it] was very good to hear that you received from VAAP the Ms of ‘Looking Back’ and started to edit the text”. In the same letter, he also states that “Of course I shall look forward to receive the edited chapters”.

And then Luria passed away on August 14, 1977.

The Coles did not desist from completing the editorial work on the autobiography. As Michael Cole wrote to one of us: “As to the editing process, I saw Luria on a visit to Moscow [probably in April 1977, see above; C.K./A.M.] and was able to confirm our editing through about three or four chapters before he died [in that very visit and/or via further correspondence, C.K./A.M.]. The remaining chapters we carried out in the same style, he had had few corrections to suggest prior to his death. However, those changes were rejections of the introduction of personal information about his life. For example, he did not want to include the fact that he was Jewish in his brief treatment of his early life” (in Métraux, 2021, pp. 182–183).

Given all the evidence provided, we now confront again the overall question: Is *The Making of Mind* a piece of Luria’s life-writing that deserves to be called (and thus classified) as a genuinely authentic autobiography?

If we argue that the revised/edited “three or four chapters” were *confirmed viva voce* and face-to-face in Cole’s presence, then at least the beginning of *The Making of Mind* may confidently be judged to be at least a fragment of Luria’s genuine authentic autobiography. The fact that two persons, using at least two different pens and an unknown number of typewriters, created parts of a text that resulted from the 1977 typescript of *Looking Back* is not a strong argument against our just-expressed conviction. Imagine that Luria had employed a ghostwriter to produce the text of the 1976 or the 1977 typescript without telling Cole or anybody else. We would still consider the first chapters of *The Making of Mind* part of a genuinely authentic autobiography because Luria had *endorsed* the Coles’ contribution(s) without reservation.

But what about the chapters of *The Making of Mind* finalised by the Coles after Luria’s death on August 14, 1977? The comparison between the 1977 typescript and the published text shows beyond any doubt that their reworking added much material to the text Luria had sent to Michael in March of that year, and that they also deleted numerous passages from the typescript. The difference between the genuinely autobiographic typescript and the edited text of *The Making of Mind* compels us to consider the latter as a partly heterobiographic contribution to a truly autobiographic project, notwithstanding the fact that Luria obviously trusted the Coles to remain faithful until the project was complete.

## Summary

Rather than summing up the results of our contribution, we list the dates of high importance in chronological order. Note that some dates are not mentioned in the text above. They complement the overall description of the history of Luria’s life-writing (Table 2).

**Table 2:** A basic chronology of Luria’s life-writing

1964	Luria writes a contribution for Edwin Boring’s *A history of psychology in autobiography*.
1973	Luria writes an autobiographic text for the *International Social Science Journal*.
1974	Luria’s contribution for *A History of Psychology in Autobiography* appears.
1973–1975	Luria writes notes for a documentary film on him that become the basis for *Looking Back*.
1976	
Febuary	Luria asks Giuseppe Cossu to translate his *Last Book* into Italian.
March 2 and 16	Luria writes to Cole and Bruner, respectively, that he is working on an autobiography. The typescripts used by the Spaniards and the Italians are dated March 1976.
March 22	Bruner writes back enthusiastically about the idea of the new book and proposes Harvard University Press (for the USA) and Open Books (GB) as publishers.
March 29	Luria suffers another more severe heart attack.
April 11	Letter from Luria to Anne-Lise Christensen, whom he sent all or part of the typescript of *Looking Back.*
May 26	Luria writes to Bruner that he used the month of his illness to type his new book. The wife of James Wertsch would revise and edit it.
Between May 26 and November 25	Bruner gets a typescript of *Looking Back* dated March 1976.
May 27	Luria tells Luciano Mecacci that *Looking Back* is ready and that Giuseppe [Cossu] is translating it into Italian.
September 15	Luria thanks Cole for his comments and proposes to postpone the publication of the book, perhaps even until after his death.
October 16	Luria writes to Cole that he would carefully reflect on his further comments. Once more he emphasises that the text is preliminary.
November 25	Bruner writes enthusiastically to Luria that he is in the middle of his autobiography.
December 5	Luria writes to Cole that it would be best if he could do the corrections with him while in Moscow.
1977	
February 14	Luria writes a letter to Michael and Sheila Cole and thanks them for their invaluable help and their comments on the book.
Spring	Renato Giunti and Maria S. Veggetti visit Luria in Moscow and talk about his autobiography.
April	Cole and Luria work together on *Looking Back* in Moscow.
April 29	VAAP sends a revised version of *Looking Back* to Cole due to Luria requesting VAAP to do so. The typescript Cole gets from VAAP is dated March 1977.
May 26	Luria writes to Bruner that he has worked with Cole on *Looking Back* in April. Once more, he expresses his desire for Bruner to write a foreword to the book.
June 8	Luria writes to Cole that it is good to have learnt that he had received a typescript of *Looking Back *from VAAP and begun to edit it.
August 14	Luria dies at the age of 75.
1978	
In the first half of 1978	Lana Pimenovna Luria dies.
April 2	The publishing house Giunti announces it will immediately stop the translation into Italian and hopes that VAAP will send them the first half of the revised book.
1978 or 1979	Nestor Bereciartu of the French publishing house Masson sends a typescript from Paris to Barcelona.
Beginning of 1979	Jordi Peña Casanova receives a typescript of *Looking Back* at Masson’s Barcelona office and translates it into Spanish together with Montserrat Pérez Pàmies.
September (or a later month) 1979	*Mirando hacia atrás* is published.
November 1st, 1979	*The Making of Mind* is published.
1982	*Etapy projdennogo puti* is published.
1983	*Uno sguardo sul passato* is published.
1985	*Itinéraires d’un psychologue* is published.
1987	*Il farsi della mente* is published.
January 20, 1992	Elena Luria dies. Copyrights for Luria’s work and archive shifts to Alexander Friedenstein (1924–1997), Elena Luria’s widower.
1992	*A construção da mente* is published.
1993	*Romantische Wissenschaft* is published.
2006	*A Dialogue with The Making of Mind* is published.
2017	The second edition of *A construção da mente* is published.
2023	The second edition of *Il farsi della mente* is published.

## Concluding Remarks and Open Questions

We have shown that Luria was active in promoting his life-writing. The task of reconstructing the overall history of *Looking Back* (from its proto-version to the publication of *The Making of Mind*) is now solved. However, some issues remain open.

We have found no evidence yet that any persons other than those mentioned in the present article were *deeply* involved in the making of *The Making of Mind*. We thus have no knowledge of the extent to which the Danish neuropsychologist Anne-Lise Christensen, who received at least parts of the 1976 typescript shortly after its completion (Christensen, 2012), offered feedback to Luria or otherwise became involved in subsequent Lurian life-writing.

Given the international orientation of Luria’s work and his many contacts with colleagues worldwide, it is possible that additional letters or other documents relating to *Looking Back* and its offspring *The Making of Mind* may at some point appear.

What is actually told in Luria’s life-writing – explicitly or between-lines – and what is omitted? This question has come up again and again, starting with Cole’s epilogue to the first American edition (Cole, 1979) and his and Karl Levitin’s efforts on the second edition intensifying such queries while reaching no conclusion (Cole & Levitin, 2006a, 2006b; see also reviews of both the first and second American editions: e.g. Bloor, 1980; Wertsch, 1980; Kozulin, 1982; Karpov, 2007).

Another question not yet dealt with concerns Luria’s concept of literary genres. In the letter of May 26, 1976 to Jerome Bruner, Luria mentioned that the subtitle of his autobiography would be “an autobiographic novel.” We do not dare to hypothesise that Luria even incidentally believed that he was producing a fictitious account vaguely inspired by his own life. Since he was quite familiar with novels, and numerous thrillers, he was probably not using the term ‘novel’ in connection with his life-writing in the technical sense of literary criticism. However, he seems to have conceived of the genre of autobiography somewhat idiosyncratically. With his scientific contributions publicly available, and thus publicly known and no longer a matter of private affairs (drafting hypotheses, collecting scientific information at home or in research institutions, planning experiments, debating issues with collaborators in non-public places, etc.), Luria could narratively summarise, contextualise, and comment in hindsight on what his *work* had yielded. The resulting autobiography was thus intended as a report on past events that had occurred in his already publicly known career. The life in various realms of the non-public, the intimate life of the otherwise public scientist was meant to remain his private affair, since it would not have been relevant, in his view, to understanding the logic behind the research practices of a Soviet psychologist, neuropsychologist, and neurolinguist. Hence the distinction between the core content of the autobiography of the public person known as Luria, on the one hand, and the non-relevant content of a novel-like narrative of the strictly private personality known to his family and friends as Alexander Romanovitch. As Michael Cole remembers, Luria basically refused to engage in narrating private things, although he often would give in and allow his editor to integrate some heterobiographic elements into descriptions of things from which the novelistic ‘private I’ would otherwise preferably have discarded.

Another aspect concerns Michael Cole’s own aims pursued in (and through) his and Sheila’s edited version as materialized in *The Making of Mind*. Did this monograph somehow ‘Americanize’ Luria and turn him into a kind of seemingly Western hero of Soviet psychology, as Lev Vygotsky *may* be perceived as having been ‘Americanized’ in *Mind in Society* (Vygotsky, 1978) by the four editors Michael Cole, Vera John-Steiner, Sylvia Scribner, and Ellen Souberman? Our answer is distinctly negative. There was – for obvious reasons of course – not the least contract or agreement between Vygotsky and either of the four editors of *Mind in Society*, whereas Luria had known, and obviously agreed to, Michael and Sheila Cole’s intentions and aims as editors of the *Making of Mind*. Hence, Luria *knew* what was going on, and the people of the Soviet copyright agency were fully aware, too, of what was going on. So that if the life-writers of Luria (Luria himself and the Coles) plus Soviet officials had intended to ‘Americanize’ the key *persona* of that book, it would have happened with a purely virtual Soviet, but actually most improbable “da” (“yes” in Russian).

The famous case studies Luria’s autobiography with heterobiographic elements turned into narrative objects do not differ formally, as already indicated above, from extended medical histories to be found in numerous publications of the late nineteenth century and of the first decades of the twentieth century. Such medical histories also occur, indeed, in Luria’s other writings (see e.g. Luria et al., 1965). However, he did not list these as instances of ‘romantic science.’

All of this leads directly to the yet-unanswered question: how does one categorise the formally different sorts of texts authored by Luria? Or in other words, how did Luria represent himself as an author of different sorts of texts?

## Data Availability

No datasets were generated or analysed during the current study.

## Footnotes

1 Sometimes “Luria” is transliterated as “Lurija”; the second transliteration is highlighted by added [ ].

2 For biographic information focusing on Luria’s time at the Kisegach war clinic see Akhutina and Pylaeva (2020/2021). Helen Proctor’s monograph also offers insights into Luria’s life, though its main focus lies on providing a social history of Luria’s research (Proctor, 2020).

3 The transcribed acronym stands for *Vsesoyuznoe agentsvo po avtorskimi pravam.*

4 It should be mentioned that Giuseppe Cossu included a translation of an excerpt from the typescript of 1976, the chapter on romantic science, as an appendix to the Italian version of Luria’s famous case study on the *Mind of a Mnemonist* (Luria [Lurija], 1979c).

5 Cf. the original typescript in http//lura.ucsd.edu/HistoryFragments.pdf; for more details see Métraux (2021, pp. 173–187).

6 Luria wrote to Jerome Bruner on June 7, 1977: “My ‘Looking Back’ is now with Michael [Cole] who is editing the text ….” (Harvard University Archive, Papers of Jerome Seymour Bruner, circa 1915–1979, HUG 4242.5 box 88.) All correspondence between Bruner and Luria quoted in the present paper stems from this source.

7 Courtesy Luciano Mecacci (personal archive).

8 Personal communication.

9 The 1976 typescript is part of the Bruner Papers located at the Harvard University Archives; the document was provided by this institution (Jerome Bruner Personal Archive. 2017.183 Box 2). In what follows we refer to it as Luria (1976c).

10
*Apprenticeship* (pp. 17–27), *Moscow* (pp. 28–37), *Vygotsky* (pp. 38–57), *Cultural differences in thinking* (pp. 58–80), *Mental development in twins* (pp. 81–103), *Verbal regulation of behavior* (pp. 104–119), *Disturbance of brain functions* (pp. 120–137), *Neuropsychology in World War II* (pp. 138–156), *Mechanisms of the brain* (pp. 157–173), *Romantic science* (pp. 174–188).

11 Rakmat also appears in the monograph on the expeditions – *Cognitive Development. Its Cultural and Social Foundations* – albeit there he is aged thirty-nine (Luria, 1976d/1974, pp. 55–58).

12 This interpretation rests on the fact that Luria also spoke in public on his experience as cultural psychologist, as a Soviet tape-recording from the last years of his life shows.

13 In another typescript (or copy of the original 1976 typescript), viz. the version Luria gave to Cossu (Luria, 1976b), the corresponding brief passage reads as “(was it Max Vesworn? )”, the lower case “n” having overwritten an equally lower case “k” (p. 237).

14 In order to avoid misunderstanding, we quote the same passage from the original publication: “*Récit rétrospectif en prose qu’une personne réelle fait de sa propre existence*,* lorsqu’elle met l’accent sur sa vie individuelle*,* en particulier sur l’histoire de sa personnalité*”.

15 On Lejeune’s approach cf. the excellent critical assessments by Allamand (2018) and Varga (2018).

16 Letter from Luria to Cole, courtesy Michael Cole. All our quotations from the correspondence between Luria and Cole stem from letters kept in Cole’s private archive.

17 See above, concerning the reason for stating that there exist three versions of two autobiographic typescripts.

18 Letter from VAAP to Cole, courtesy Michael Cole.

## Declarations

### Letters

Correspondence M. Casini (Giunti) – M. Cole and VAAP, 20.4.1978. Private Archive Michael Cole (San Diego).

Correspondence A. R. Luria – P. Blake, 10.12.1975. Private Archive Michael Cole (San Diego).

Correspondence A. R. Luria – J. Bruner, 1976–1977. Harvard University Archives, Jerome Bruner Papers, General Correspondence 1975–1977, HUG 4242.5, Box 88 (Cambridge, MA).

Correspondence A. R. Luria – M. Cole (and S. Cole), 1975–1977. Private Archive Michael Cole (San Diego).

Correspondence A. R. Luria – L. Mecacci, 27.5.1976. Private Archive Luciano Mecacci (Florence).

Correspondence VAAP – M. Cole, 29.4.1977. Private Archive Michael Cole (San Diego).

Correspondence V. Zinchenko – Cole, 1979. Private Archive Michael Cole (San Diego).

### Personal Communication

Personal communication took place via email, virtual meetings and/or telephone with the following persons in 2023 and 2024:

Tatiana Akhutina (Moscow).

Michael Cole (San Diego).

Giuseppe Cossu (Padua).

Luciano Mecacci (Florence).

Jordi Peña Casanova (Barcelona).

Maria Serena Veggetti (Rome).

James Wertsch (St. Louis).
