# Correction: Multiple Lurias. Reconstructing Alexander Romanovich’s Life-Writing

**DOI:** 10.1007/s12124-025-09898-4

**Published:** 2025-02-14

**Authors:** Carlos Kölbl, Alexandre Métraux

**Affiliations:** 1 Lehrstuhl für Psychologie, Kulturwissenschaftliche Fakultät, Universität Bayreuth, 95440 Bayreuth, Germany; 2 Archives Henri Poincaré, Université de Lorraine, Nancy, France

**Integrative Psychological and Behavioral Science (2025) 59:21**

10.1007/s12124-024-09872-6

The original version of this article unfortunately contained mistakes.

p. 6, top line, Khomskaya’s should be Homskaya’s.

p. 6, 5th paragraph, (Luria, 1979a; Lurija, 1983) should read (Luria, 1979a; Luria [Lurija] 1983)

p. 8, 4th paragraph, [Looking Back, 1976, p. 6]) should read [Looking Back, 1976c, p. 6])

p. 10, 9th line, (Looking Back 1976, pp. 6–7) should read (Looking Back 1976c, pp. 6–7).

p. 15, 3rd paragraph, (ibid.; see also Fig. 1 below) should read (ibid.; see also Fig. 1 above).

p. 17, top line, A passage of the 1977 typescript reads thus should read A passage of the 1977 typescript which is identical to the corrected passage of the 1976 typescript (see Fig. 1) reads thus.

p. 17, 1st paragraph, the sentence In this disease the pathological state of the subcortical motor ganglia disturb the voluntary flow of movement so deeply that in a very short time, less than a minute, movement becomes impossible should read In this disease the pathological state of the subcortical motor ganglia disturb the involuntary flow of movement so deeply that in a very short time, less than one minute, movement becomes impossible.

Reference Cossu, G. (1987). Uomini che vanno e idee che restano. In A. R. Lurija, M. and S. Cole, introduction and epilogue by M. Cole, preface and translation by G. Cossu, (Eds.), Il farsi della mente: Autobiografia di A. R. Lurija. (pp. I–V). Rome: Armando Editore should be changed to Cossu, G. (1987). Uomini che vanno e idee che restano. In A. R. Lurija, Il farsi della mente: Autobiografia di A. R. Lurija (pp. I–V; Eds.: M. and S. Cole, introduction and epilogue by M. Cole, preface and translation by G. Cossu). Rome: Armando Armando.

Reference Luria [Lurija], E. A. (1994). Moj otec A. R. Lurija [My father A. R. Luria]. Gnozis should read Luria [Lurija], E. A. (1994). Moj otec A. R. Lurija [My father A. R. Luria]. Moscow.

Reference Luria, A. R. (1976d). Cognitive development. In M. Cole (Ed.), Its cultural and social foundations. Cambridge/MA: Harvard University. (Original work published in 1974) should read Luria, A. R. (1976d). Cognitive development. Its cultural and social foundations. Cambridge/MA: Harvard University. (Original work published in 1974).

Reference Métraux, A. (2021). Alexander Luria: Marxist psychologist and transnational scientific broker. A personal account. In A. Yasnitsky (Ed.), A history of marxist psychology. The golden age of soviet science (pp. 156–190). Routledge should read Métraux, A. (2021). Alexander Luria: Marxist psychologist and transnational scientific broker. A personal account. In A. Yasnitsky (Ed.), A history of marxist psychology. The golden age of soviet science (pp. 156–190). London and New York: Routledge.

Reference Proctor, H. (2020). Psychologies in revolution. Alexander Luria’s ‘Romantic science’ and soviet social history. Palgrave Macmillan should read Proctor, H. (2020). Psychologies in revolution. Alexander Luria’s ‘Romantic science’ and soviet social history. Cham: Palgrave Macmillan.

Referecne Van der Veer, R., & Valsiner, J. (1991). Understanding Vygotsky. A quest for synthesis. Blackwell should read Van der Veer, R., & Valsiner, J. (1991). Understanding Vygotsky. A quest for synthesis. Oxford: Blackwell.

Reference Varga, Z. Z. (2018). About the contractual nature of the autobiographical pact. In T. G. Ashplant, C. Brant, & I. Luca (Eds.), Cher Philippe. A festschrift for Philippe Lejeune on the occasion of his 80th birthday (pp. 57–65). Panchaud should read Varga, Z. Z. (2018). About the contractual nature of the autobiographical pact. In T. G. Ashplant, C. Brant, & I. Luca (Eds.), Cher Philippe. A festschrift for Philippe Lejeune on the occasion of his 80th birthday (pp. 57–65). Amsterdam: Panchaud.

Reference Veggetti, S. M. (1983). Presentazione al lettore italiano. In A. R. Lurija, & S. M. Veggetti (Eds.), Uno sguardo sul passato. Considerazioni retrospettive sulla vita di uno psicologo sovietico (pp. V–X). Florence: Giunti should read Veggetti, S. M. (1983). Presentazione al lettore italiano. In A. R. Lurija (Eds.), Uno sguardo sul passato. Considerazioni retrospettive sulla vita di uno psicologo sovietico (pp. V-X; Ed.: S. M. Veggetti). Florence: Giunti.

The original article has been updated.

